# The Novel Coronavirus Enigma: Phylogeny and Analyses of Coevolving Mutations Among the SARS-CoV-2 Viruses Circulating in India

**DOI:** 10.2196/20735

**Published:** 2020-09-07

**Authors:** Anindita Banerjee, Rakesh Sarkar, Suvrotoa Mitra, Mahadeb Lo, Shanta Dutta, Mamta Chawla-Sarkar

**Affiliations:** 1 Indian Council of Medical Research-National Institute of Cholera and Enteric Diseases Kolkata India

**Keywords:** SARS-CoV-2, Indian isolates, phylogeny, nucleotide homology, novel coevolving mutations, NSP12/RdRP secondary structure, genetics, virus, evolution, mutation, COVID-19, genome, epidemiology, infectious disease

## Abstract

**Background:**

The RNA genome of the emerging novel coronavirus is rapidly mutating, and its human-to-human transmission rate is increasing. Hence, temporal dissection of their evolutionary dynamics, the nature of variations among different strains, and understanding the single nucleotide polymorphisms in the endemic settings are crucial. Delineating the heterogeneous genomic constellations of this novel virus will help us understand its complex behavior in a particular geographical region.

**Objective:**

This is a comprehensive analysis of 95 Indian SARS-CoV-2 genome sequences available from the Global Initiative on Sharing All Influenza Data (GISAID) repository during the first 6 months of 2020 (January through June). Evolutionary dynamics, gene-specific phylogeny, and the emergence of the novel coevolving mutations in 9 structural and nonstructural genes among circulating SARS-CoV-2 strains across 12 different Indian states were analyzed.

**Methods:**

A total of 95 SARS-CoV-2 nucleotide sequences submitted from India were downloaded from the GISAID database. Molecular Evolutionary Genetics Analysis, version X software was used to construct the 9 phylogenetic dendrograms based on nucleotide sequences of the SARS-CoV-2 genes. Analyses of the coevolving mutations were done in comparison to the prototype SARS-CoV-2 from Wuhan, China. The secondary structure of the RNA-dependent RNA polymerase/nonstructural protein NSP12 was predicted with respect to the novel A97V mutation.

**Results:**

Phylogenetic analyses revealed the evolution of “genome-type clusters” and adaptive selection of “L”-type SARS-CoV-2 strains with genetic closeness to the bat severe acute respiratory syndrome–like coronaviruses. These strains were distant to pangolin or Middle East respiratory syndrome–related coronavirus strains. With regard to the novel coevolving mutations, 2 groups have been seen circulating in India at present, the “major group” (66/95, 69.4%) and the “minor group” (21/95, 22.1%) , harboring 4 and 5 coexisting mutations, respectively. The “major group” mutations fall in the A2a clade. All the minor group mutations, except 11083G>T (L37F, NSP6 gene), were unique to the Indian isolates.

**Conclusions:**

This study highlights the rapidly evolving SARS-CoV-2 virus and the cocirculation of multiple clades and subclades. This comprehensive study is a potential resource for monitoring the novel mutations in the viral genome, interpreting changes in viral pathogenesis, and designing vaccines or other therapeutics.

## Introduction

The COVID-19 pandemic caused by the novel SARS-CoV-2 was initially reported from Wuhan, China in December 2019, but it spread across the world within 3 months [1]. As of July 21, 2020, more than 14.9 million people have been found to be infected by SARS-CoV-2, with a death toll of approximately 615,939 in more than 210 countries. Phylogenetic analyses reveal that SARS-CoV-2 clusters within the subgenus *Sarbecovirus* under the genus *Betacoronavirus* and has probably undergone zoonotic transmission from the bats through the possible intermediate host Malayan pangolins, culminating among humans [2]. The positive sense, single-stranded RNA genome of SARS-CoV-2 is continuously mutating and generating multiple clades within a short time span (December 2019 to June 2020). Hence, there is a need to dissect the complex evolutionary characteristics of this novel coronavirus, identifying the single nucleotide polymorphisms (SNPs) and other mutations among strains circulating across different parts of the world. Previous reports on the genetic and evolutionary dynamics of the SARS-CoV-2 virus have tried to deduce the mode of transmission that this virus made its way into humans from bats during the early phase of the pandemic, but many questions remain unanswered even though more sequence data has been made available. Therefore, studying the heterogeneous genomic constellations within specific geographical settings will help to understand its complex epidemiology and formulate region specific strategies to curb its spread and severity.

The first 3 cases from India with travel history to Wuhan were reported in Kerala during January 2020 [3]; subsequently, 4,02,529 active cases, 724,577 recovered cases, and 28,084 deaths have been officially recorded in India as of July 21, 2020, 8 AM India Standard Time GMT +5:30 [4]. India ranks third worldwide according to the number of COVID-19 infections and is geographically vulnerable to this novel virus, as it accounts for almost 6% of global and 3.5% of COVID-19–attributable mortality. In spite of high population density, poor hygiene conditions, and an overburdened health care system, the proportion of the total infected population is much lower when compared to other western countries, that is, 0.05% in India versus 0.87% in the United States, 0.73% in Brazil, 0.46% in Russia, and 0.4% in Italy. Though the average death rate due to SARS-CoV-2 infection in India (2.46%) is comparable to that of world (eg, the United States 3.88%, Europe 6.6%), 50% of deaths in India are attributable to the age group 40-64 years [5]. Thus, to understand the phylodynamics of circulating strains in India, this study was initiated to analyze the complete viral genome sequences submitted in the Global Initiative on Sharing All Influenza Data (GISAID) [6] from 95 SARS-CoV-2 representative strains circulating across 12 differentially affected states within India. To elucidate the possible ancestry, gene-wise phylogeny of these Indian strains has been deciphered with respect to other isolates reported from Europe, the United States, and China along with coronavirus strains belonging to other genera infecting humans and other animal hosts. The novel coevolving mutations among the Indian SARS-CoV-2 strains have also been analyzed.

Through this genome analyses and phylogenetic approach, we have attempted to focus on the natural evolution of SARS-CoV-2 from its existing ancestors within the zoonotic reservoir. Furthermore, analyzing the novel mutations accumulated within the viral genome over the period with reference to the Wuhan strains (clade O) will underscore their impact on the structure and function of viral proteins.

## Methods

### Sequence Mining

A total of 95 SARS-CoV-2 nucleotide sequences submitted from India from January to June 2020 were downloaded from the GISAID database for phylogenetic analyses and screening of novel mutations. Several other reference gene sequences of SARS-CoV-2 as well as other types of coronaviruses were downloaded from the GenBank database submitted from several other countries for dendrogram construction and further lineage analyses.

### Phylogenetic Analyses and Screening of Mutations

Nine phylogenetic dendrograms were constructed with respect to 2 structural genes (spike and nucleocapsid) and 7 nonstructural genes (nonstructural protein [NSP]2, NSP3, NSP4, NSP6, NSP7, NSP8, and NSP12). Multiple sequence alignment for all the respective set of gene sequences was done using MUSCLE v3.8.31 (drive5). Amino acid sequences were deduced through TRANSEQ (EMBL-EBI). Phylogenetic dendrograms were constructed by Molecular Evolutionary Genetics Analysis, version X (MEGAX), using the maximum-likelihood statistical method (at 1000 bootstrap replicates) and using the best fit nucleotide substitution models for each dendrogram. The best fit models were determined through model testing parameter of MEGAX. Different novel coexisting mutations in the Indian strains were identified and analyzed in comparison to the prototype SARS-CoV-2 strain from Wuhan (MN908947.3/SARS-CoV-2 Wuhan-Hu-1).

### Secondary Structure Prediction of RNA-Dependent RNA Polymerase Having A97V Mutation

We used the Chou and Fasman Secondary Structure Prediction (CFSSP) online server to predict the secondary structure of RNA-dependent RNA polymerase (RdRP)/NSP12 with novel A97V mutation [7].

## Results

### Phylogenetic Analysis of the Structural and Nonstructural Genes

#### Spike Gene

Among the 95 Indian study isolates, 93 strains clustered among themselves within the same lineage of *Betacoronavirus* SARS-CoV-2 in 3 different subclusters (A=53, B=12, and C=28 strains), while 2 strains, 1 from Telangana (EPI_ISL_ 431101) and the other from Maharashtra (EPI_ISL_ 479550), extruded out separately, close to subcluster C containing the clade-specific strains A1, A3, B1, B2, B4-1, and B4-2 in the phylogenetic dendrogram for the spike (S) gene. The prototype SARS-CoV-2 strain belonging to the O clade (MN908947.3/SARS-CoV-2/HUMAN/CHN/Wuhan-Hu-1/2019) was present in the same lineage with the Indian strains within subcluster C (>99% identity). Subcluster A comprised of the clade-specific A2 and A2a strains along with a tiger strain from the New York zoo and another carnivorous mammal, mink SARS-CoV-2. No specific pattern of temporal distribution of strains was observed among the 3 subclusters. All the representative Indian strains had 99%-100% nucleotide sequence homology among themselves. The Indian strains had 92.8%-93% and 83.5% homology with Bat (EPI_ISL_402131 /COV /BAT /YUNNAN /RATG13 /2013) and Pangolin coronavirus (EPI_ISL_410540 /COV /PANGOLIN /GUANGXI / P5L/2017), respectively. Homology was much less (75.8%-76.7%) with other bat severe acute respiratory syndrome (SARS)–like coronavirus strains (eg, MG772933.1/ SARS-LIKE-COV/BAT/ BAT-SL-COVZC45 /2017 and MG772934.1/ SARS-LIKE-COV/ BAT/ BAT-SL-COVZXC21 /2015), while Middle East respiratory syndrome–related coronavirus (MERS-CoV; KJ713299.1 /MERS-COV /CAMEL /SAU /KSA-CAMEL-376 /2013 and KU308549.1/MERS-COV/ HUMAN/ KOR/ SEOUL-SNU1-035/ 2015) were distantly related to the Indian SARS-CoV-2 strains (52.5%-52.9% identity; Multimedia Appendix 1).

#### Nucleocapsid Gene

The phylogenetic dendrogram for the nucleocapsid (N) gene revealed that out of 95 Indian study isolates, 92 strains clustered within the same lineage of *Betacoronavirus* SARS-CoV-2 in 3 different subclusters (A=33 strains, B=47 strains, and C=12 strains), while 3 strains from Tamil Nadu (EPI_ISL_ 458040), Gujarat (EPI_ISL_ 458107), and Delhi (EPI_ISL_ 435111) extruded out separately, close to subcluster C strains. Subcluster A comprised of strains from the earlier 3 months (January, February, and March), while subcluster B contained strains from the later 3 months. Subcluster C had mixed strains. The clade-specific strains (A1, A1a, A2, A2a, A5, B1, and B4-1) as well as the prototype SARS-CoV-2 strain O clade (MN908947.3/SARS-CoV-2/HUMAN/CHN/Wuhan-Hu-1/2019) clustered near subcluster B, while B4-2, A3, and B2 clade strains were close to subcluster C. All the representative Indian strains had >99.8% nucleotide identity among themselves as well as with the different clade-specific strains. The Indian strains had 91%-97% sequence identity with bat coronaviruses (EPI_ISL_402131 /COV/BAT /YUNNAN /RATG13 /2013, MG772933.1 /SARS-LIKE-COV /BAT /BAT-SL-COVZC45 /2017, and MG772934.1 /SARS-LIKE-COV /BAT/ BAT-SL-COVZXC21/ 2015) and 91% similarity with pangolin strains (EPI_ISL_410540 /COV /PANGOLIN/GUANGXI/ P5L /2017). In contrast to bat strains, the MERS-CoV strains (KJ713299.1/MERS-COV/CAMEL /SAU /KSA-CAMEL-376 /2013 and KU308549.1/MERS-COV/HUMAN /KOR /SEOUL-SNU1-035/2015) were genetically distant to the Indian SARS-CoV-2 strains (56.9%-57.3% identity; Multimedia Appendix 2).

#### RNA-Dependent RNA Polymerase Gene (RdRP/NSP12)

The phylogenetic dendrogram for the RdRP/NSP12 gene depicted that, among the 95 Indian study isolates, 93 strains clustered within the same lineage of *Betacoronavirus* SARS-CoV-2 into 3 subclusters (A=64 strains, B=22 strains, and C=7 strains). Two strains, one from Kerala (EPI_ISL_ 413523) and the other from Delhi (EPI_ISL_ 435111), were placed distant to these 3 subclusters in the dendrogram and were close to A1a, A2, A3, A5, B1, B2, B4-1, B4-2, and the prototype O clade strains. Subcluster A strains clustered with the A2a clade-specific strain while subcluster C clustered with A1. No temporal specificity was observed among the 3 subcluster strain distributions. All the Indian strains had >99.8% nucleotide identity among themselves as well as the different clade-specific strains. The prototype SARS-CoV-2 strain O clade (MN908947.3 /SARS-COV-2 /HUMAN /CHN /Wuhan-Hu-1 /2019) was distant to all the 3 subclusters. The Indian strains had 97.8% sequence homology with bat coronavirus (EPI_ISL_402131 /COV /BAT /YUNNAN /RATG13/2013) and 86.7%-88.6% similarity with both pangolin (EPI_ISL_410540 /COV /PANGOLIN /GUANGXI/ P5L/2017) and other bat SARS-like coronavirus strains (MG772933.1 /SARS-LIKE-COV /BAT /BAT-SL-COVZC45 /2017 and MG772934.1 /SARS-LIKE-COV /BAT /BAT-SL-COVZXC21/2015). MERS-CoV strains (KJ713299.1 /MERS-COV /CAMEL /SAU/ KSA-CAMEL-376/2013 and KU308549.1 /MERS-COV /HUMAN /KOR /SEOUL-SNU1-035 /2015) were distantly related to the Indian SARS-CoV-2 strains (68.1% identity; Multimedia Appendix 2).

#### NSP2, NSP3, NSP4, NSP6, NSP7, and NSP8 Genes

The dendrograms of all these 6 genes showed a similar pattern. All the 95 Indian strains clustered in 2 subclusters (A=39 and B=56 strains) within the *Betacoronavirus* lineage of SARS-CoV-2. Principally, subcluster A strains were from the first 3 months, whereas B contained strains from the next 3 months of 2020. Strains of subcluster A and B had 99.9%-100% DNA homology among themselves. All the clade-specific strains (A1, A1a, A2, A2a, A3, A5, B1, B2, B4-1, and B4-2) along with the prototype SARS-CoV-2 strain clade O (MN908947.3/SARS-CoV-2 /HUMAN /CHN /Wuhan-Hu-1 /2019) clustered close to subcluster A (99.9% identity), except NSP7 and NSP8 where the prototype clade O strain was present within subcluster B strains. SARS-CoV-2 strains isolated from carnivorous mammals like mink and tiger also grouped close to the subcluster A strains in all the dendrograms (99.9% identity). Subcluster A and B strains revealed 95.4%-98.1% nucleotide sequence similarity with bat coronavirus EPI_ISL_402131 /COV /BAT /YUNNAN /RATG13 /2013, while the pangolin-derived strain EPI_ISL_410540/COV/PANGOLIN/GUANGXI/P5L/2017 showed less identity (83%-87.5%). MERS-CoV strains NC_019843.3/MERS-COV/HUMAN/NLD/HCOV-EMC/2012 and KU740200.1 /MERS-COV /CAMEL /EGYPT /NRCE-NC163/2014 exhibited a significant phylogenetic distance (only 49.6%-60.8% homology) from the Indian isolates (Multimedia Appendicies 4-9).

### L- and S-Type of SARS-CoV-2

SNPs at positions 8782 (NSP4 gene) and 28,144 (open reading frame [ORF]8) showed complete linkage among the Indian isolates under study. At these two sites, 93 strains showed a “CT” haplotype (designated as “L” type as T28,144 falls in the codon position which encodes amino acid leucine in the 84th position of ORF8 protein), while only 2 strains (1 from Kerala, EPI_ISL_413523, and 1 from Delhi, EPI_ISL_435111) revealed a “TC” haplotype (called as “S” type as C28,144 falls in the codon encoding serine at the 84th position of the ORF8 protein).

### Analyses of Synonymous and Nonsynonymous Mutations

#### The Common Mutations in SARS-CoV-2 Indian Isolates

To explore the mutations among the 95 SARS-CoV-2 strains, we performed in-depth sequence analyses both at the genome level and at the corresponding amino acid level in different proteins, especially S glycoprotein, N protein, NSP2, NSP3, NSP4, NSP6, NSP7, NSP8, and RdRP/NSP12 with reference to the prototype SARS-CoV-2 strain (MN908947.3/SARS-CoV-2/HUMAN/CHN/Wuhan-Hu-1/2019). Out of 95 samples, 2 (2.1%) were found to have no significant “L”-type mutations (EPI_ISL_435111 and EPI_ISL_413523). Out of 93 “L”-type samples, 6 (6.3%; EPI_ISL_481156, EPI_ISL_476840, EPI_ISL_476023, EPI_ISL_458080, EPI_ISL_431101, and EPI_ISL_413522) harbored none of the mutations and were wild-type–like. Mutational analysis of the remaining 87 strains revealed circulation of two predominant “groups,” namely, the “major group” and the “minor group,” across India. The “major group,” which, of 95 isolates, was comprised of 66 (69.4%), revealed 4 coexisting SNPs: 241C>T in the five prime untranslated region (5′ UTR), 3037C>T (F106F) in the NSP3 gene, 14408C>T (P323L) in the NSP12 gene, and 23403A>G (D614G) in the S gene (Table 1). This “major group” of SARS-CoV-2 was predominantly found to circulate in regions like Delhi, Maharashtra, West Bengal, Odisha, Telangana, and Gujarat. The other 21 (22.1%) samples, which represent the “minor group,” harbored 5 coexisting mutations: 23929C>T (Y789Y) in the S gene, 28311C>T (P13L) in the N gene, 6312C>A (T1198K) in the NSP3 gene, 11083G>T (L37F) in the NSP6 gene, and 13730C>T (A97V) in the NSP12/RdRP gene (Table 2). Needless to say, the 5 coexisting mutations of the “minor group” and the 4 coexisting mutations of the “major group” did not overlap among the same SARS-CoV-2 strains. The “minor group” of SARS-CoV-2 predominated across Tamil Nadu (South) and Uttar Pradesh (Central/North).

**Table 1:** Single nucleotide polymorphisms associated with the major group SARS-CoV-2 strains (n=66) across India from January to June 2020.^a^

State and accession number			Spike glycoprotein (21,563-25,384 nts/1273 amino acids)									RdRP^b^ protein (13,442-16,236 nts/932 amino acids)			NSP^c^3 protein (2720-8554 nts/1945 amino acids)							5’-UTR^d^ (1-265 nts)/noncoding			N^e^ protein (28,274-29,533 nts/419 amino acids)									NSP2 protein (806-2719 nts/638 amino acids)
			Q271R^f^		D614G^g^		G1124V^h^		D294D^i^		P323L^j^			F106F^k^			A994D^l^		K1249K^m^		241C>T			S194L^n^			RG203KR^o^		R41R^p^		T393I^q^		T85I^r^	
**Delhi (n=13)**																																		
	EPI_ISL_ 435061, EPI_ISL_ 435062, EPI_ISL_ 482665 (n=3)			✓						✓			✓							✓														
	EPI_ISL_ 482498 (n=1)			✓						✓			✓							✓			✓											
	EPI_ISL_ 435065-EPI_ISL_ 435069 (n=5)			✓				✓		✓			✓							✓			✓											
	EPI_ISL_ 435070, EPI_ISL_ 435071 (n=2)			✓						✓			✓							✓						✓								
	EPI_ISL_ 435063, EPI_ISL_ 435064 (n=2)			✓						✓			✓							✓												✓		
**Tamil Nadu (n=4)**																																		
	EPI_ISL_ 458032, EPI_ISL_ 458033, EPI_ISL_ 458044, EPI_ISL_ 458040 (n=4)			✓						✓			✓							✓														
**Maharashtra (n=13)**																																		
	EPI_ISL_ 479493, EPI_ISL_ 479510, EPI_ISL_ 479553, EPI_ISL_ 479533, EPI_ISL_ 479538, EPI_ISL_ 479497, EPI_ISL_ 479550, EPI_ISL_ 479554, EPI_ISL_ 479557, EPI_ISL_ 479560, EPI_ISL_ 479562, EPI_ISL_ 479571, EPI_ISL_ 479564 (N=13)			✓						✓			✓			✓				✓														
**West Bengal (n=5)**																																		
	EPI_ISL_ 430466, EPI_ISL_ 430467 (n=2)			✓						✓			✓							✓														
	EPI_ISL_ 430465 (n=1)			✓						✓			✓							✓						✓								
	EPI_ISL_ 430468, EPI_ISL_ 430464 (n=2)			✓		✓				✓			✓							✓						✓								
**Gujarat (n=11)**																																		
	EPI_ISL_ 458107, EPI_ISL_ 483878, EPI_ISL_ 476869, EPI_ISL_ 469036, MT576031 (n=5)			✓						✓			✓							✓														
	EPI_ISL_ 461484, EPI_ISL_ 476864 (n=2)			✓				✓		✓			✓							✓			✓											
	EPI_ISL_ 471637 (n=1)			✓						✓			✓							✓						✓								
	EPI_ISL_ 475058 (n=1)			✓						✓			✓			✓				✓						✓								
	EPI_ISL_ 426414, EPI_ISL_ 426415 (n=2)	✓		✓						✓			✓					✓		✓								✓		✓				
**Odisha (n=7)**																																		
	EPI_ISL_ 481154, EPI_ISL_ 481157 (n=2)			✓						✓			✓							✓														
	EPI_ISL_ 481115, EPI_ISL_ 463078, EPI_ISL_ 481177, EPI_ISL_ 481180, EPI_ISL_ 481186 (n=5)			✓						✓			✓							✓						✓								
**Madhya Pradesh (n=2)**																																		
	EPI_ISL_ 476884, EPI_ISL_ 476842 (n=2)			✓						✓			✓							✓														
**Telangana (n=5)**																																		
	EPI_ISL_ 458080, EPI_ISL_ 431101 (n=2)			✓						✓			✓							✓														
	EPI_ISL_ 431117 (n=1)			✓						✓			✓							✓			✓											
	EPI_ISL_ 471588 (n=1)			✓				✓		✓			✓							✓			✓											
	EPI_ISL_ 471629 (n=1)			✓						✓			✓							✓						✓								
**Karnataka (n=5)**																																		
	EPI_ISL_ 477207, EPI_ISL_ 477250 (n=2)			✓						✓			✓							✓														
	EPI_ISL_ 477255, EPI_ISL_ 477237, 477239 (n=3)			✓				✓		✓			✓							✓			✓											
**Uttar Pradesh (n=1)**																																		
	EPI_ISL_ 435060 (n=1)			✓						✓			✓							✓														

^a^Mutations were analyzed with compared to Wuhan-Hu-1 (MN908947.3).

^b^RdRP: RNA-dependent RNA polymerase.

^c^NSP: nonstructural protein.

^d^5′ UTR: five prime untranslated region.

^e^N: nucleocapsid.

^f^22374A>G.

^g^23403A>G.

^h^24933G>T.

^i^22444C>T.

^j^14408C>T.

^k^3037C>T.

^l^5700C>A.

^m^6466A>G.

^n^28854C>T.

^o^28881-28883 GGG>AAC.

^p^28396G>A.

^q^29451C>T.

^r^1059C>T

**Table 2:** Single nucleotide polymorphisms associated with the minor group SARS-CoV-2 strains (n=21) across India during January to June 2020.^a^

State and accession number		Spike glycoprotein (21,563-25,384 nts/1273 amino acids)	RdRP^b^ protein (13,442-16,236 nts/932 amino acids)	N^c^ protein (28,274-29,533 nts/419 amino acids)	NSP^d^3 protein (2720-8554 nts/1945 amino acids)		NSP6 protein (10,973-11,842 nts/290 amino acids)
		Y789Y (23929C>T)	A97V (13730C>T)	P13L (28311C>T)	S1197R (6310C>A)	T1198K (6312C>A)	L37F (11083G>T)
**Tamil Nadu (n=8)**							
	EPI_ISL_435093- EPI_ISL_435096, EPI_ISL_435084, EPI_ISL_435087 (n=6)	✓	✓	✓		✓	✓
	EPI_ISL_435091, EPI_ISL_435092 (n=2)	✓	✓	✓	✓	✓	✓
**Maharashtra (n=1)**							
	EPI_ISL_435077 (n=1)	✓	✓	✓		✓	✓
**Odisha (n=2)**							
	EPI_ISL_463017 (n=1)	✓	✓	✓		✓	✓
	EPI_ISL_463010 (n=1)	✓	✓	✓	✓	✓	✓
**Madhya Pradesh (n=1)**							
	EPI_ISL_476848 (n=1)	✓	✓	✓		✓	✓
**Telangana (n=1)**							
	EPI_ISL_431103 (n=1)	✓	✓	✓		✓	✓
**Karnataka (n=4)**							
	EPI_ISL_486399, EPI_ISL_486394, EPI_ISL_486408, EPI_ISL_MT396248 (n=4)	✓	✓	✓		✓	✓
**Uttar Pradesh (n=3)**							
	EPI_ISL_435100, EPI_ISL_435099, EPI_ISL_435082 (n=3)	✓	✓	✓		✓	✓
**Bihar (n=1)**							
	EPI_ISL_435112 (n=1)	✓	✓	✓		✓	✓

^a^Mutations were analyzed with compared to Wuhan-Hu-1 (MN908947.3).

^b^RdRP: RNA-dependent RNA polymerase.

^c^N: nucleocapsid.

^d^NSP: nonstructural protein.

#### The Unique Mutations in SARS-CoV-2 Indian Isolates

In addition to 23403A>G (D614G), 3 uncommon mutations, 23374A>G (Q271R), 24933G>T (G1124V), and 22444C>T (D294D), were also observed in the S gene of the “major group” (Table 1). Out of the 67 isolates of the major group, 28 revealed 4 novel mutations: 28854C>T (S194L; n=13), 28881-28883GGG>AAC (R203K and G204R; n=13), and coevolving mutation 29451C>T (T393I) and 28395G>A (R41R; n=2) in the N gene (Table 1). Intriguingly, 28854C>T (S194L) in the N gene was found to coevolve with the 22444C>T (D294D) mutation in the S gene of 11 samples in the major group (Table 1). We also observed 1059T>A (T85I) change within the NSP2 gene (n=2) and 6466A>G (K1249K) change in the NSP3 gene (n=2). With the 3 samples of the minor group, 6310C>A (S1197R) was found to be associated. No mutations were found within the NSP7 and NSP8 genes.

#### Effect of Missense Mutation A97V on the Secondary Structure of NSP12/RdRP

RdRP is the crucial enzyme for both viral RNA replication and maintenance of genomic fidelity. Thus, any significant change in RdRP structure could affect its functions, leading to an increase in the rate of mutagenesis in the genome. We have identified 2 missense mutations in the RdRP protein: P323L associated with the “major group” isolates and A97V associated with the “minor group” isolates. The effect of P323L on the secondary structure of RdRP has already been described [8]. Therefore, we analyzed the effect of novel mutation A97V on the secondary structure of RdRP by using the CFSSP server. The A97V mutation resulted in substitution of α-helixes at positions 94, 95, and 96 within the β-sheets in the RdRP secondary structure, which may alter its tertiary conformation and affect functionality (Multimedia Appendix 10).

## Discussion

### Principal Findings

The molecular and genetic characterization of SARS-CoV-2 pandemic strains worldwide has been studied by several scientific groups based on whole-genome sequencing [9,10]. Through this comprehensive analysis, we aimed to closely investigate the ancestry, evolutionary dynamics, accumulation of rapid mutations, and cross-genetic translation among the emerging SARS-CoV-2 strains across India. Rapid accumulation of several point mutations across the genome of SARS-CoV-2 since its origin is a prime driving force behind the evolution of different monophyletic clades. As depicted through the different phylogenetic dendrograms in our study, a monophyletic clade of all SARS-CoV-2 strains was seen with the prototype strain (Wuhan IME-WH01/2019). Clustering of all the Indian isolates with other SARS-CoV-2 strains reported worldwide (99.8%-100% nucleotide sequence identity) suggests the introduction of this virus in India was from several countries. The clustering pattern of the prototype strain from Wuhan in the phylogenetic dendrogram underscores the fact that China might have served as the origin of this zoonotic virus, which was eventually transmitted worldwide [11-13].

The origin of SARS-CoV-2 is still undetermined, but identification of its intermediate host is much needed to prevent further dissemination and interspecies transmission in the near future. Hence, we initiated this study as one of the first in India to decipher the gene-wise phylogenetics of SARS-CoV-2 strains circulating in this endemic setting. The results depicted genome-type clusters of the 95 Indian isolates, for the structural genes S and N, and the nonstructural gene RdRP/NSP12. Clustering of the study isolates with different clade-specific strains for different genes established the development of genome-type clustering. Though variations in DNA homology exists with respect to each gene, a recent bifurcation of these SARS-CoV-2 strains from the bat- and Malayan pangolin–derived SARS-like coronaviruses is supposed to have occurred, with a subsequent zoonotic transmission to humans, as depicted through all 9 dendrograms. Moreover, the SARS-CoV-2 strains were distant to MERS-CoV and other human coronaviruses. This conclusion goes at par with other phylogenetic studies establishing bat and pangolins as the proximal origin of SARS-CoV-2 [14-16].

Our study highlighted the low sequence similarity of the S gene of the Indian study isolates with some bat-derived strains like bat-SL-CoVZC45 and bat-SL-CoVZXC21, while maximum homology was noticed with bat SARS-like coronavirus (SARSr-CoV/RaTG13). This observation was consistent with a report where the S gene of SARS-CoV-2 strains circulating within China revealed the lowest sequence homology (nearly 70%) with bat strains (like SL-CoVZC45 and SL-CoVZXC21), in comparison to 96.2% identity to bat SARS-related coronavirus (SARSr-CoV/RaTG13). The RNA-binding domain within the S1 subunit of the S gene of all Indian SARS-CoV-2 and pangolin-derived strains were found to be evolutionarily conserved and phylogenetically much closer than bat RaTG13, underscoring the familiar mode of pathogenesis between the two. The Indian SARS-CoV-2 isolates too possess a polybasic cleavage site (RRAR; amino acid position 682-685) at the junction of S1 and S2 subunits of the S protein as reported by Andersen et al [14]. SARS-CoV-2 strains have been categorized into two major groups or types characterized by two SNPs at positions 8782 (NSP4 gene) and 28,144 (ORF8) that reveal complete linkage [17]. Among our Indian study isolates, frequency of the L-type (CT haplotype) was much higher (93/95, 97.9%) to the S-type (TC haplotype; 2/95, 2.1%), indicating the predominance of L-type over S-type in this geographical region.

Convoluted mutational analysis also revealed cocirculation of 2 groups of mutated SARS-CoV-2 strains in India. The “major group” of SARS-CoV-2 strains (66/95, 69.4%) represents the A2a clade reported previously from Africa, South America, Oceania, and South and West Asia, comprising of strains with coevolving mutations like 241 C>T (5′ UTR), 3037 C>T (F106F, NSP3), 14403 C>T (P323L, RdRP/NSP12), and 23403 A>G (D614G, S glycoprotein) [18-20]. Certain strains in the “major group” displayed 22374A>G (Q271R), 24933G>T (G1124V), and 22444C>T (D294D) changes in the S gene, which were unique to India. Missense mutations, Q271R and G1124V in the S protein, were found to reside around the N-linked glycosylation sites 282 and 1134, respectively, and these might affect the protein function [21]. It was not surprising to observe the triple site mutation 28881-28883 GGG>AAC (R203K and G204R) in the N gene of 13 SARS-CoV-2 strains of the “major group.” This has previously been reported from Mexico, South America, Australia, New Zealand, and a few Asian countries [22]. The 203/204 region is part of the SR dipeptide domain of the N protein (*SR*NS*SR*NSTPGS*SR*GTSPARMA) and changes in arginine at position 203 to lysine; and glycine at position 204 to arginine resulted in the insertion of a lysine residue between serine and arginine (*SR*NS*SR*NSTPGS*SKR*TSPARMA), which might interfere with the phosphorylation at serine residue required for normal functioning of the N protein [23]. This mutation demands particular attention as reduced pathogenicity has been observed previously in SARS-related coronavirus on deletion of the SR domain [24]. Mutations observed in the NSP3 gene at positions 6310 C>A (S1197R), 7392 C>T (P1558L), and 6466 A>G (K1249K) were completely unique to Indian strains. Few infrequent mutations at position 1059 T>A (T85I) in NSP2 and 8782 C>T (S76S) in NSP4 observed here have also been reported to be prevalent in other countries [19,22,25].

The “minor group” of Indian SARS-CoV-2 (21/95, 22.1%) was comprised of strains with 5 coevolving mutations: 13730C>T (A97V, RdRP/NSP12), 23929C>T (Y789Y, S), 28311C>T (P13L, N), 6312C>A (T1198K, NSP3), and 11083G>T (L37F, NSP6). All the “minor group” mutations were novel among the Indian isolates, except 11083G>T (L37F, NSP6), which was previously reported as an infrequent mutation from Australia, Japan, Netherlands, and some other European countries [18,26]. The L37F mutation strongly implies positive selection toward evolution of *Betacoronaviruses*, indicating a possible origin of the “minor group” out of this positive selection, with subsequent acquisition of mutations among the strains already harboring the 11083G>T change [25,26]. The interaction of NSP6 with NSP3 and NSP4 has been described to be essential for the formation of double membrane vesicles [25,26]. Hence, it is interesting to note the presence of a coexisting mutation 6312 C>A (T1198K) in NSP3 of the “minor group” strains, though the significance of this coexistence (L37F and T1198K) in context to the NSP6-NSP3 interaction can only be confirmed through association studies. The functional accuracy of RdRP is challenged due to the presence of the 13730 C>T (A97V) change, which was predicted to have significant effect on the secondary structure of RdRP [8]. In addition, A97V was found to be located in the nidovirus RdRP-associated nucleotidyl transferase domain whose function remains unknown [27]. The P13L mutation is located in the intrinsically disordered region of the N protein and might affect RND-binding activity of the N-terminal domain and C-terminal domain of the N protein [28,29].

Any significant mutation in the RdRP/NSP12 protein might alter replication machinery, thereby compromising the fidelity of viral RNA replication and subsequent accumulation of plausible novel mutations. The missense mutation 14408C>T (P323L) in RdRP was first observed in Italy (Lombardy) in February 2020. Few strains from Europe and North America since February 2020 have shown the emergence of mutations like 3037C>T (F106F, NSP3), 23403A>G (D614G), and 28881-28883GGG>AAC (R203K and G204R, N) in the SARS-CoV-2 genome harboring the 14408C>T (P323L) mutation within the RdRP gene, suggesting a probable association or coexistence of 14408C>T (P323L) and the emerging higher number of novel point mutations compared to viral genomes from Asia [30]. Therefore, we can assume that two mutations, 14408C>T (P323L) and 13730C>T (A97V), which were found to have significant influence on the secondary structure of RdRP, could play key roles in the simultaneous establishment of “two groups” of SARS-CoV-2 with several characteristic “co-evolving mutations” in India (Asia). However, this needs to be validated experimentally. A recent study reported that the frequency of mutations within the SARS-CoV-2 genome varies in different geographical areas, as SARS-CoV-2 gene sequences from Europe and North America present an overwhelming mutation frequency compared to that of Asia. Their study identified few recurrent mutations among isolates from Europe that were not detected among the viruses circulating within Asian countries, such as 3037C>T (F106F/NSP3 gene), 14408C>T (P323L/RdRP gene), 28881-28883GGG>AAC (R203K and G204R/N gene), and 23403A>G (D614G/S gene) [30]. In contrast, our analyses revealed that all these mutations accumulated over time beyond Europe and were profoundly seen among the “major group” of SARS-CoV-2 strains circulating across India (Asia).

The free availability of genome sequences in the publicly available servers like National Center for Biotechnology Information and GISAID has revolutionized the genome studies, resulting in continuous monitoring of mutations, recombination events, development of molecular diagnostics, identification of vaccine strains, etc. The ongoing deadly pandemic requires recording the complete patient metadata along with full genome sequences of the SARS-CoV-2 strains for better understanding of the epidemiology and virulence of this virus. Exploiting newer technologies that could help in recording additional information such as specific disease traits (comorbidity, respiratory scores, essential blood parameters), treatment, requirement of hospitalization or outpatient treatment, treatment outcome, life-threatening complication, or mortality in addition to the full viral genome sequences. This would also help in geographical region-based decisions regarding treatment modalities as well as inclusion of highly virulent subtypes of strains in vaccine formulations.

### Conclusion

India harbors a greater risk of community transmission of COVID-19 due to high population density, a large population below the poverty line, and overburdened health care facilities. Hence, stringent surveillance and monitoring of the viral epidemiology and genetic diversity of a novel virus can pave way for better health care strategies and vaccine designing. This study provides comprehensive analysis of the ancestry, evolutionary dynamics, clade-specific genetic variations, as well as development of unique coevolving mutations among SARS-CoV-2 strains circulating across different regions in India. Owing to the lack of patient metadata, the impact of novel mutations on the clinical outcome or the difference in virulence of the two distinct groups of circulating strains in India could not be determined.

## Appendix

Multimedia Appendix 1 Molecular phylogenetic analysis by the maximum likelihood method. Phylogenetic dendrogram based on nucleotide sequences of S gene of SARS-CoV-2 strains circulating in India during early 2020, with other known strains of respective genotype. The representative Indian strains have been marked with a solid circle. The scale‐bar was set at 0.1 nucleotide substitutions per site. Bootstrap values of less than 70% are not shown. The best fit model, which was used for constructing the phylogenetic dendrogram, was the general time reversible model with gamma distribution having invariant sites (GTR+G+I). S: spike.

Multimedia Appendix 2 Molecular phylogenetic analysis by maximum likelihood method. Phylogenetic dendrogram based on nucleotide sequences of N gene of SARS-CoV-2 strains circulating in India during early 2020, with other known strains of respective genotype. The representative Indian strains have been marked with a solid circle. Scale‐bar was set at 0.1 nucleotide substitutions per site. Bootstrap values of less than 70% are not shown. The best fit model, which was used for constructing the phylogenetic dendrogram, was the general time reversible model with gamma distribution having invariant sites (GTR+G+I). N: nucleocapsid.

Multimedia Appendix 3 Molecular phylogenetic analysis by maximum likelihood method. Phylogenetic dendrogram based on nucleotide sequences of RdRP/NSP12 gene of SARS-CoV-2 strains circulating in India during early 2020, with other known strains of respective genotype. The representative Indian strains have been marked with a solid circle. Scale‐bar was set at 0.1 nucleotide substitutions per site. Bootstrap values of less than 70% are not shown. The best fit model, which was used for constructing the phylogenetic dendrogram, was the general time reversible model with gamma distribution (GTR+G). NSP12: nonstructural protein 12; RdRP: RNA-dependent RNA polymerase.

Multimedia Appendix 4 Molecular phylogenetic analysis by maximum likelihood method. Phylogenetic dendrogram based on nucleotide sequences of the NSP2 gene of SARS-CoV-2 strains circulating in India during early 2020, with other known strains of respective genotype. The representative Indian strains have been marked with a solid circle. Scale‐bar was set at 0.1 nucleotide substitutions per site. Bootstrap values of less than 70% are not shown. The best fit model, which was used for constructing the phylogenetic dendrogram, was the general time reversible model with gamma distribution (GTR+G). NSP2: nonstructural protein 2.

Multimedia Appendix 5 Molecular phylogenetic analysis by maximum likelihood method. Phylogenetic dendrogram based on nucleotide sequences of the NSP3 gene of SARS-CoV-2 strains circulating in India during early 2020, with other known strains of respective genotype. The representative Indian strains have been marked with a solid circle. Scale‐bar was set at 0.1 nucleotide substitutions per site. Bootstrap values of less than 70% are not shown. The best fit model, which was used for constructing the phylogenetic dendrogram, was the General Time Reversible model (GTR). NSP3: nonstructural protein 3.

Multimedia Appendix 6 Molecular phylogenetic analysis by maximum likelihood method. Phylogenetic dendrogram based on nucleotide sequences of NSP4 gene of SARS-CoV-2 strains circulating in India during early 2020, with other known strains of respective genotype. The representative Indian strains have been marked with a solid circle. Scale‐bar was set at 0.1 nucleotide substitutions per site. Bootstrap values of less than 70% are not shown. The best fit model, which was used for constructing the phylogenetic dendrogram, was the general time reversible model having invariant sites (GTR+I). NSP4: nonstructural protein 4.

Multimedia Appendix 7 Molecular phylogenetic analysis by maximum likelihood method. Phylogenetic dendrogram based on nucleotide sequences of the NSP6 gene of SARS-CoV-2 strains circulating in India during early 2020, with other known strains of respective genotype. The representative Indian strains have been marked with a solid circle. Scale‐bar was set at 0.1 nucleotide substitutions per site. Bootstrap values of less than 70% are not shown. The best fit model, which was used for constructing the phylogenetic dendrogram, was the Tamura-3 model having invariant sites (T92+I). NSP6: nonstructural protein 6.

Multimedia Appendix 8 Molecular phylogenetic analysis by maximum likelihood method. Phylogenetic dendrogram based on nucleotide sequences of NSP7 gene of SARS-CoV-2 strains circulating in India during early 2020, with other known strains of respective genotype. The representative Indian strains have been marked with a solid circle. Scale‐bar was set at 0.1 nucleotide substitutions per site. Bootstrap values of less than 70% are not shown. The best fit model, which was used for constructing the phylogenetic dendrogram, was the Tamura-3 model with gamma distribution (T92+G). NSP7: nonstructural protein 8.

Multimedia Appendix 9 Molecular phylogenetic analysis by maximum likelihood method. Phylogenetic dendrogram based on nucleotide sequences of NSP8 gene of SARS-CoV-2 strains circulating in India during early 2020, with other known strains of respective genotype. The representative Indian strains have been marked with a solid circle. Scale‐bar was set at 0.1 nucleotide substitutions per site. Bootstrap values of less than 70% are not shown. The best fit model, which was used for constructing the phylogenetic dendrogram, was the general time reversible model with gamma distribution having invariant sites (GTR+G+I). NSP8: nonstructural protein 8.

Multimedia Appendix 10 Effect of A97V mutation on the secondary structure of RdRP/NSP12 protein. (A) Secondary structure of RdRP around 97th A (Alanine) residue of Wuhan isolate of SARS-CoV-2. (B) Secondary structure of RdRP around 97th V (Valine) residue of Indian isolate of SARS-CoV-2. NSP12: nonstructural protein 12; RdRP: RNA-dependent RNA polymerase.

## Abbreviations

CFSSP: Chou and Fasman Secondary Structure Prediction
GISAID: Global Initiative on Sharing All Influenza Data
MEGAX: Molecular Evolutionary Genetics Analysis, version X
MERS-CoV: Middle East respiratory syndrome–related coronavirus
N: nucleocapsid
NSP: nonstructural protein
ORF: open reading frame
RdRP: RNA-dependent RNA polymerase
S: spike
SARS: severe acute respiratory syndrome
SNP: single nucleotide polymorphism
5′ UTR: five prime untranslated region
