# Supplementary figures and images for: The Novel Coronavirus Enigma: Phylogeny and Analyses of Coevolving Mutations Among the SARS-CoV-2 Viruses Circulating in India
Source: JMIR Bioinform Biotechnol. 2020 Sep 7;1(1):e20735. doi: 10.2196/20735 (PMC7720937; doi:10.2196/20735)

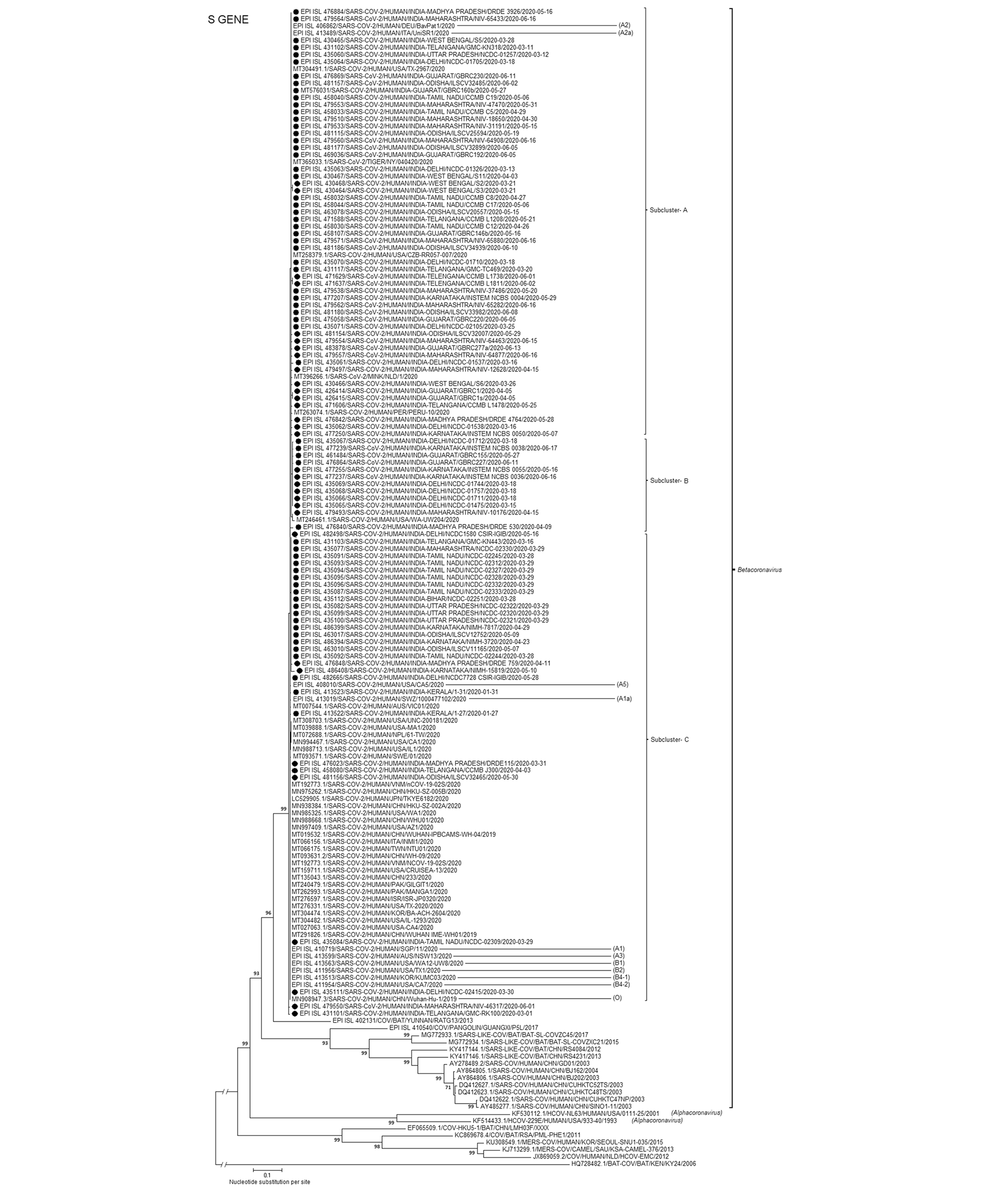

Supplement: Multimedia Appendix 1 [file bioinform_v1i1e20735_app1.png]

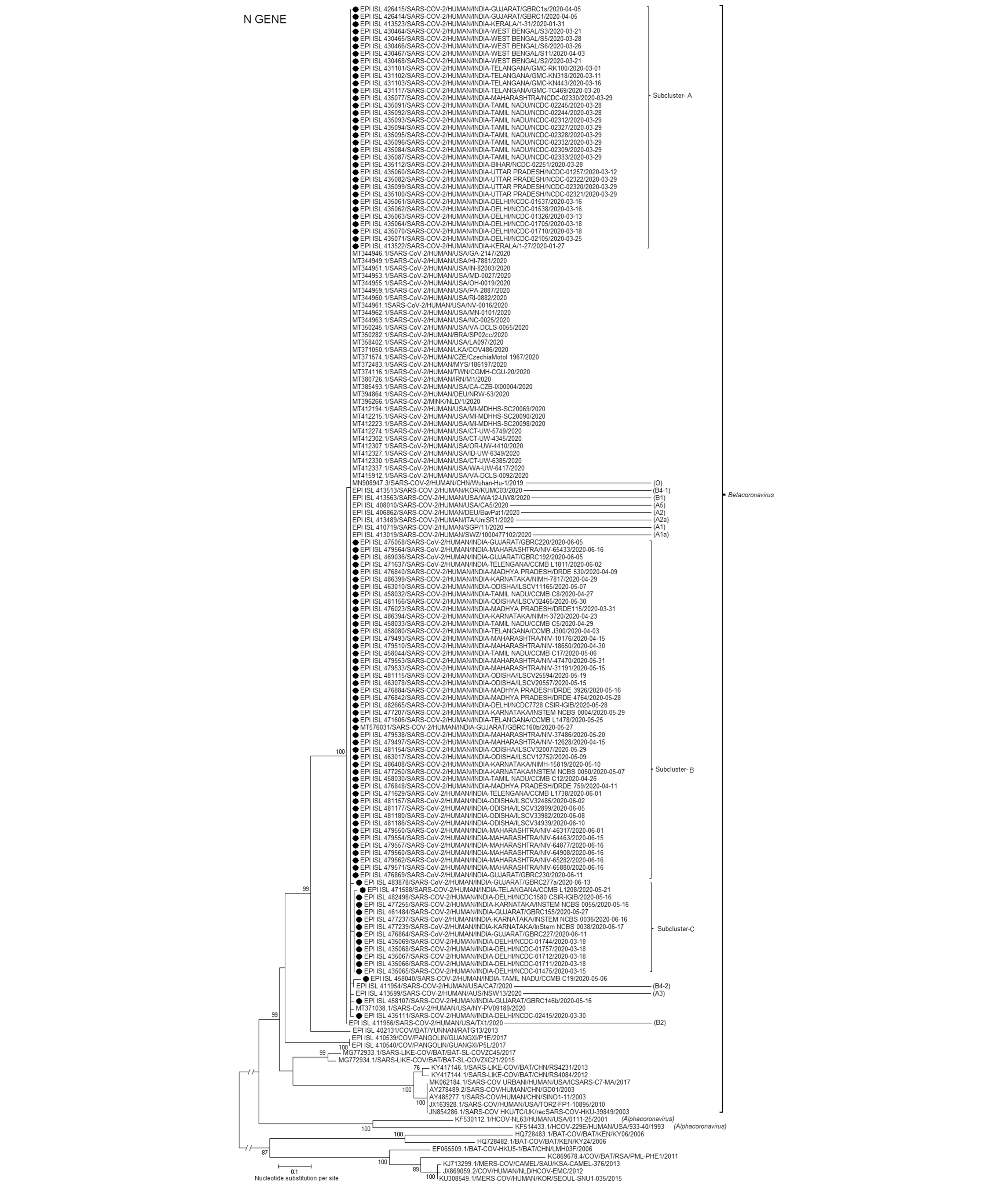

Supplement: Multimedia Appendix 2 [file bioinform_v1i1e20735_app2.png]

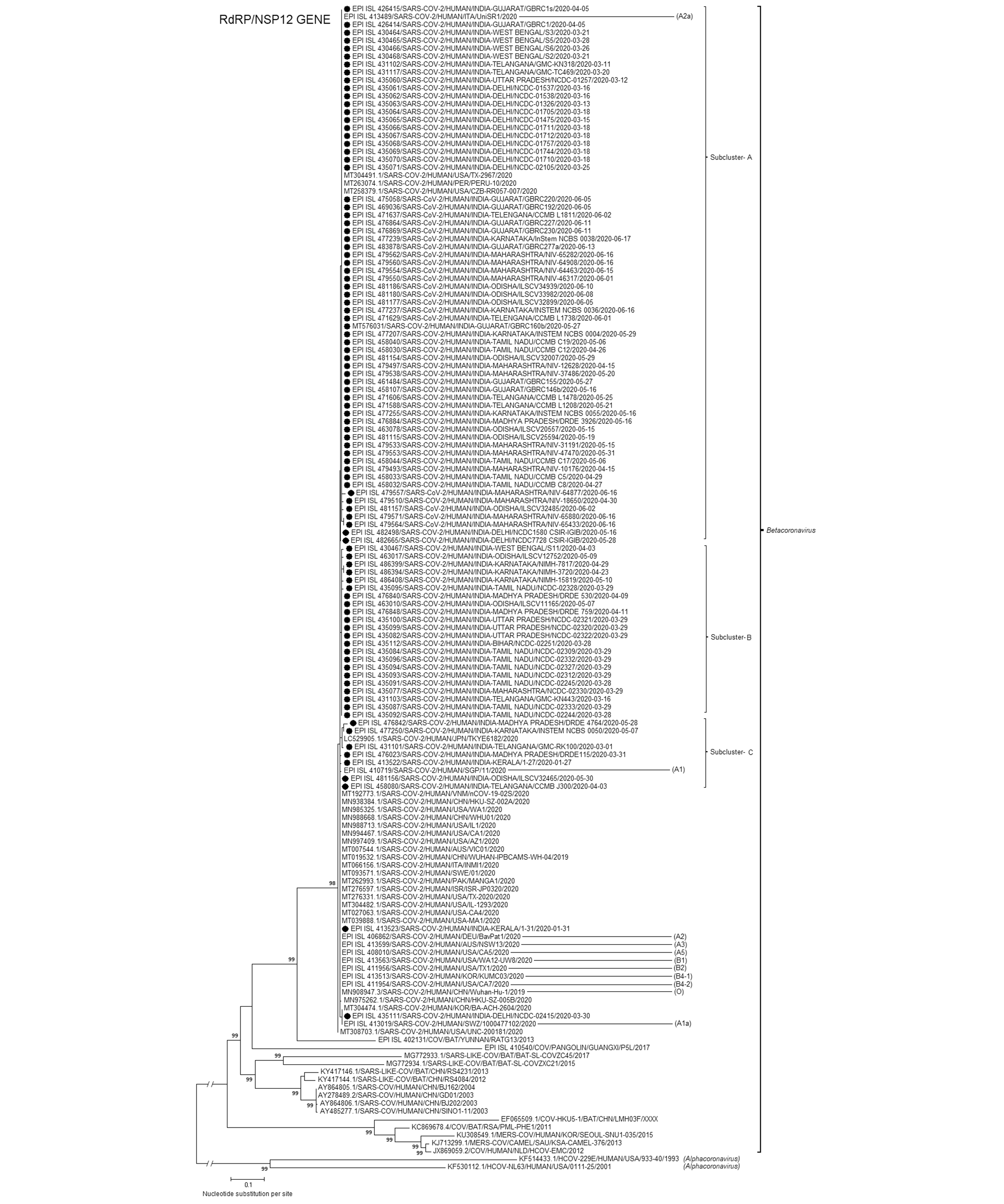

Supplement: Multimedia Appendix 3 [file bioinform_v1i1e20735_app3.png]

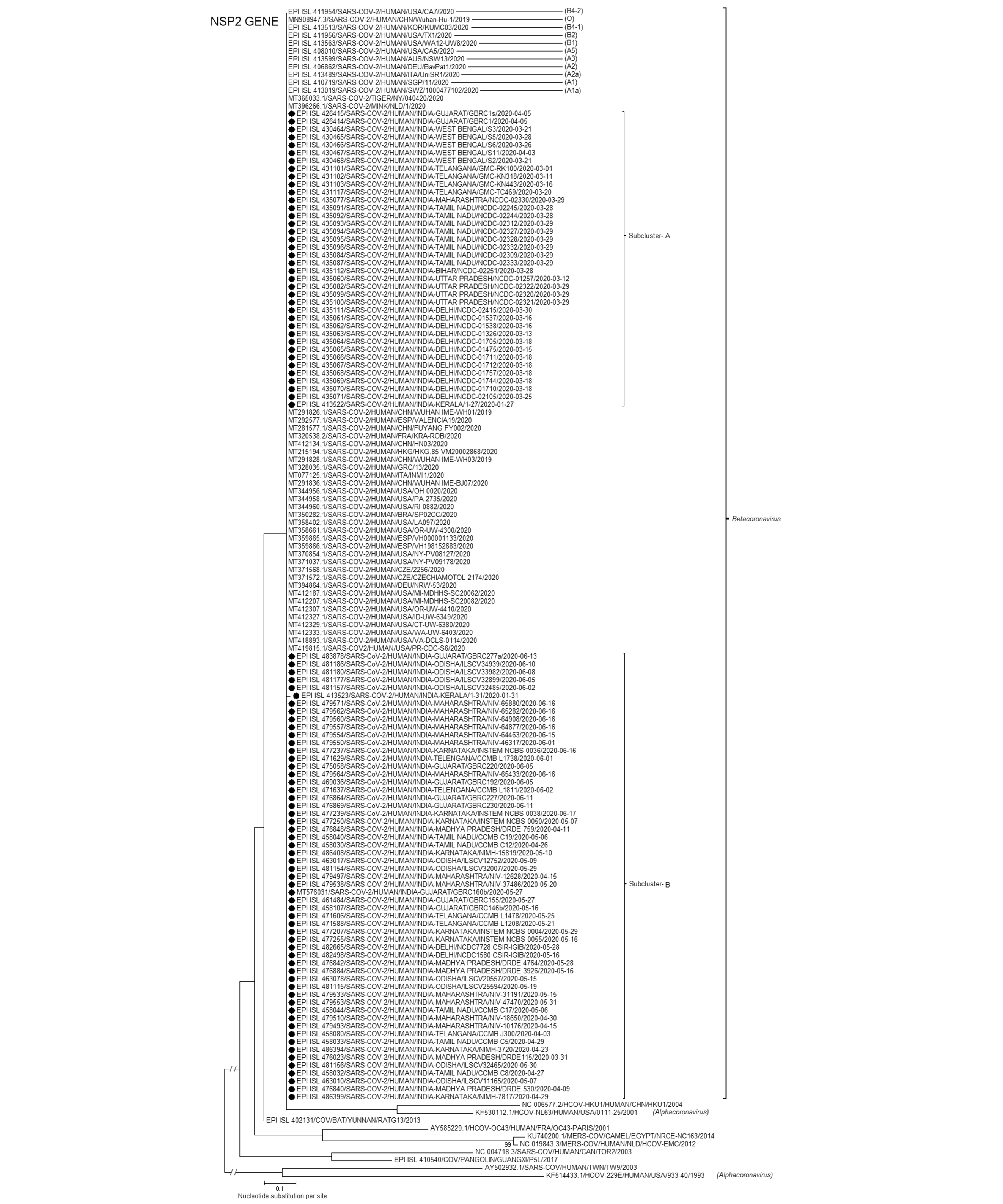

Supplement: Multimedia Appendix 4 [file bioinform_v1i1e20735_app4.png]

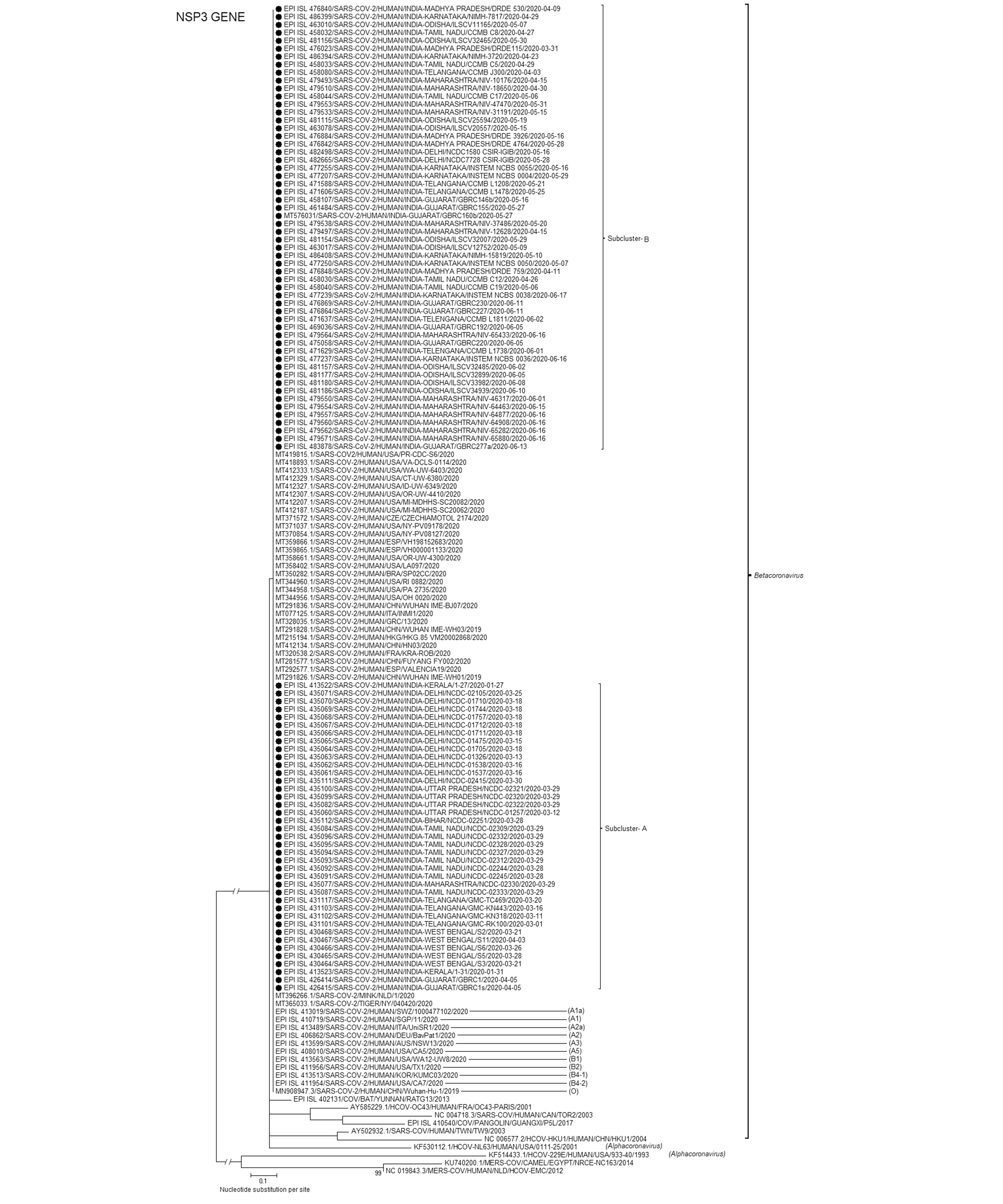

Supplement: Multimedia Appendix 5 [file bioinform_v1i1e20735_app5.png]

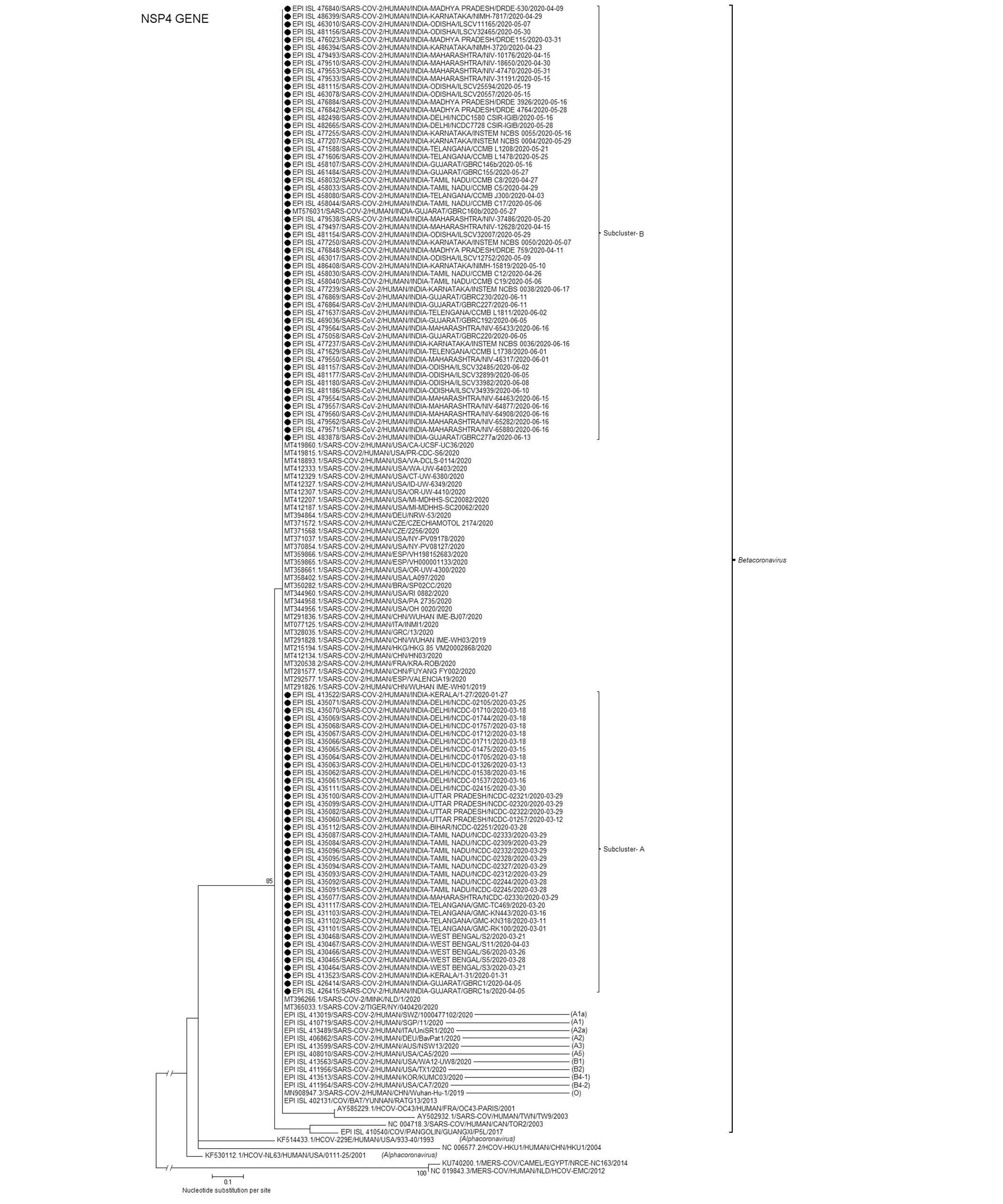

Supplement: Multimedia Appendix 6 [file bioinform_v1i1e20735_app6.png]

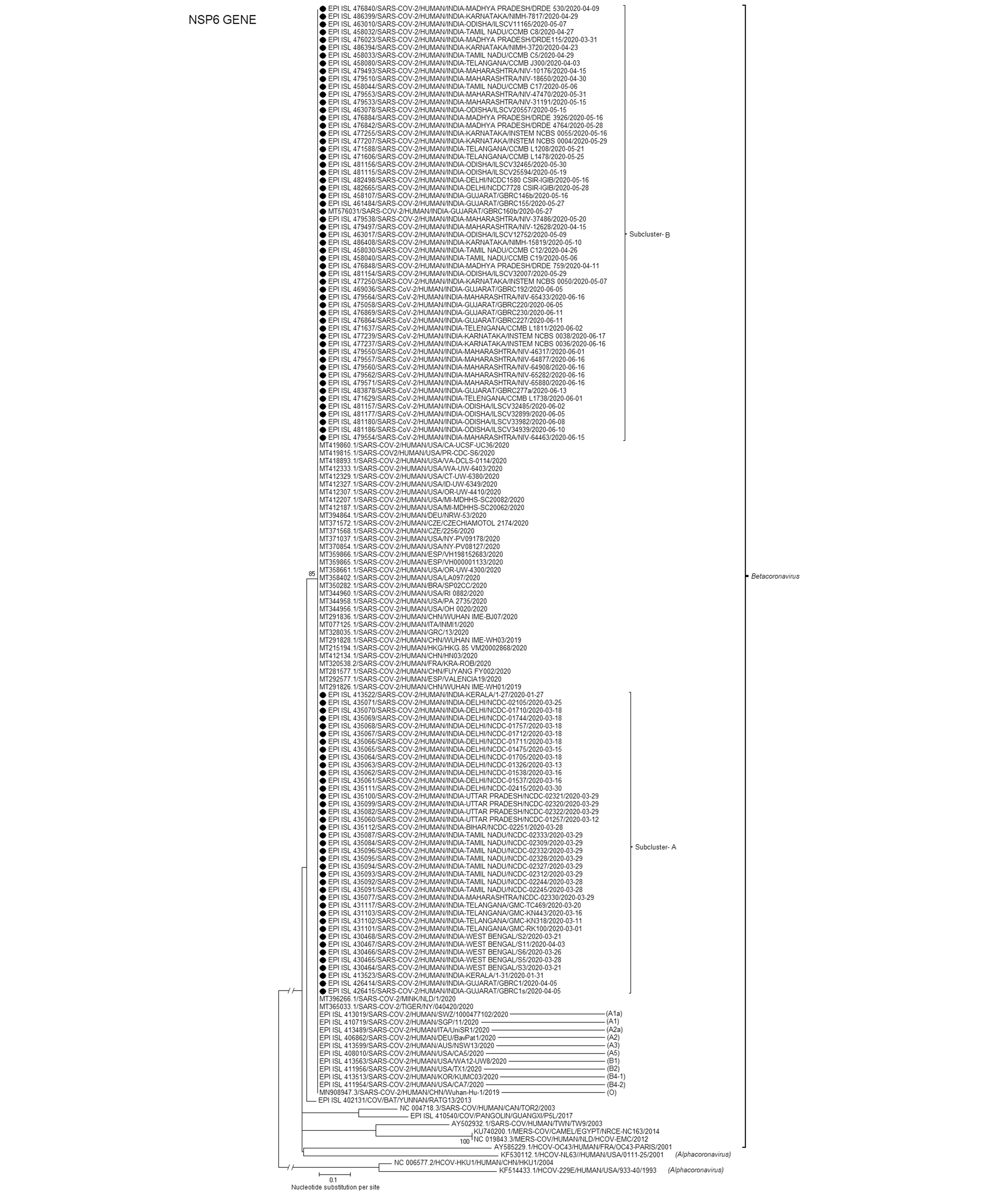

Supplement: Multimedia Appendix 7 [file bioinform_v1i1e20735_app7.png]

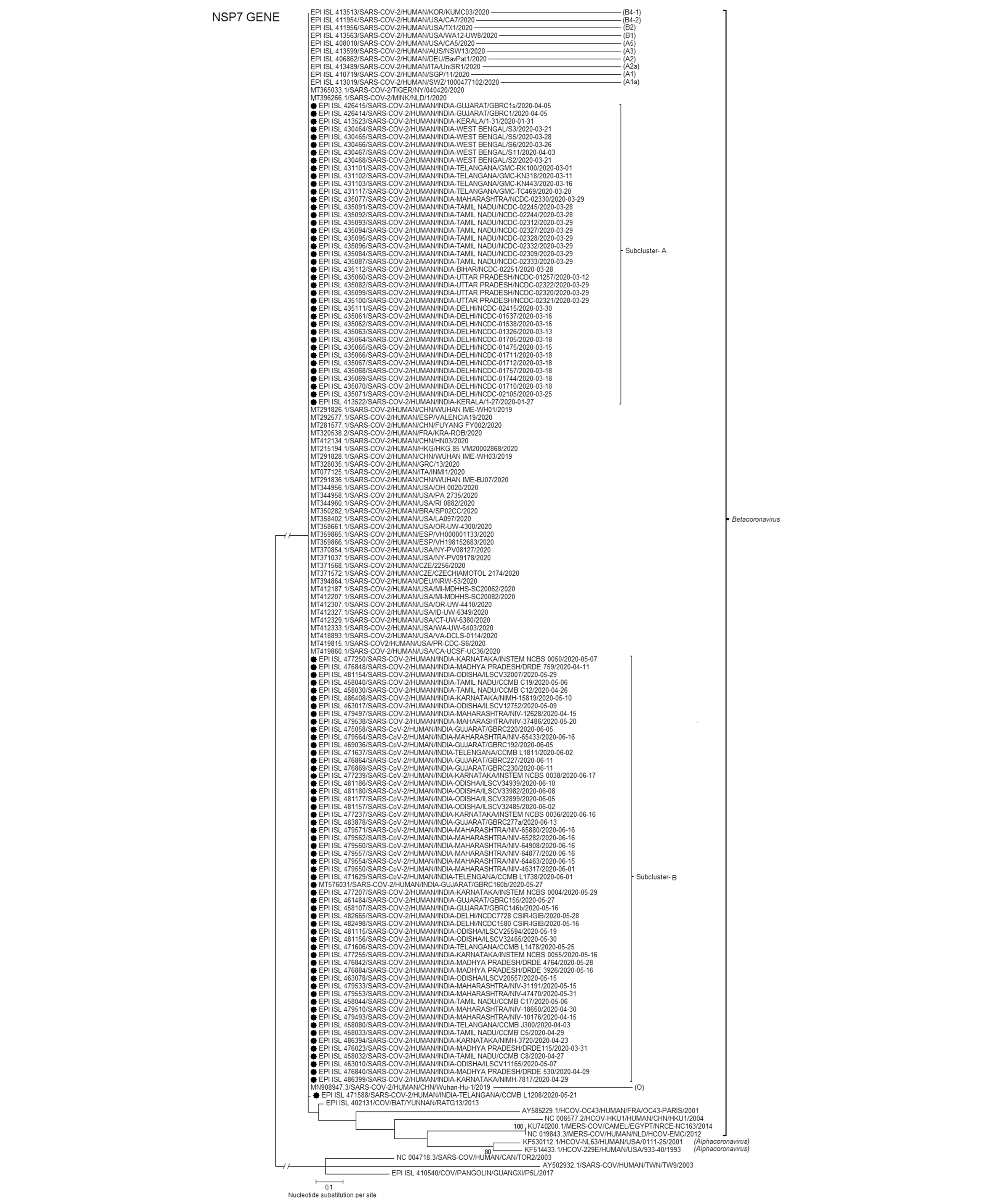

Supplement: Multimedia Appendix 8 [file bioinform_v1i1e20735_app8.png]

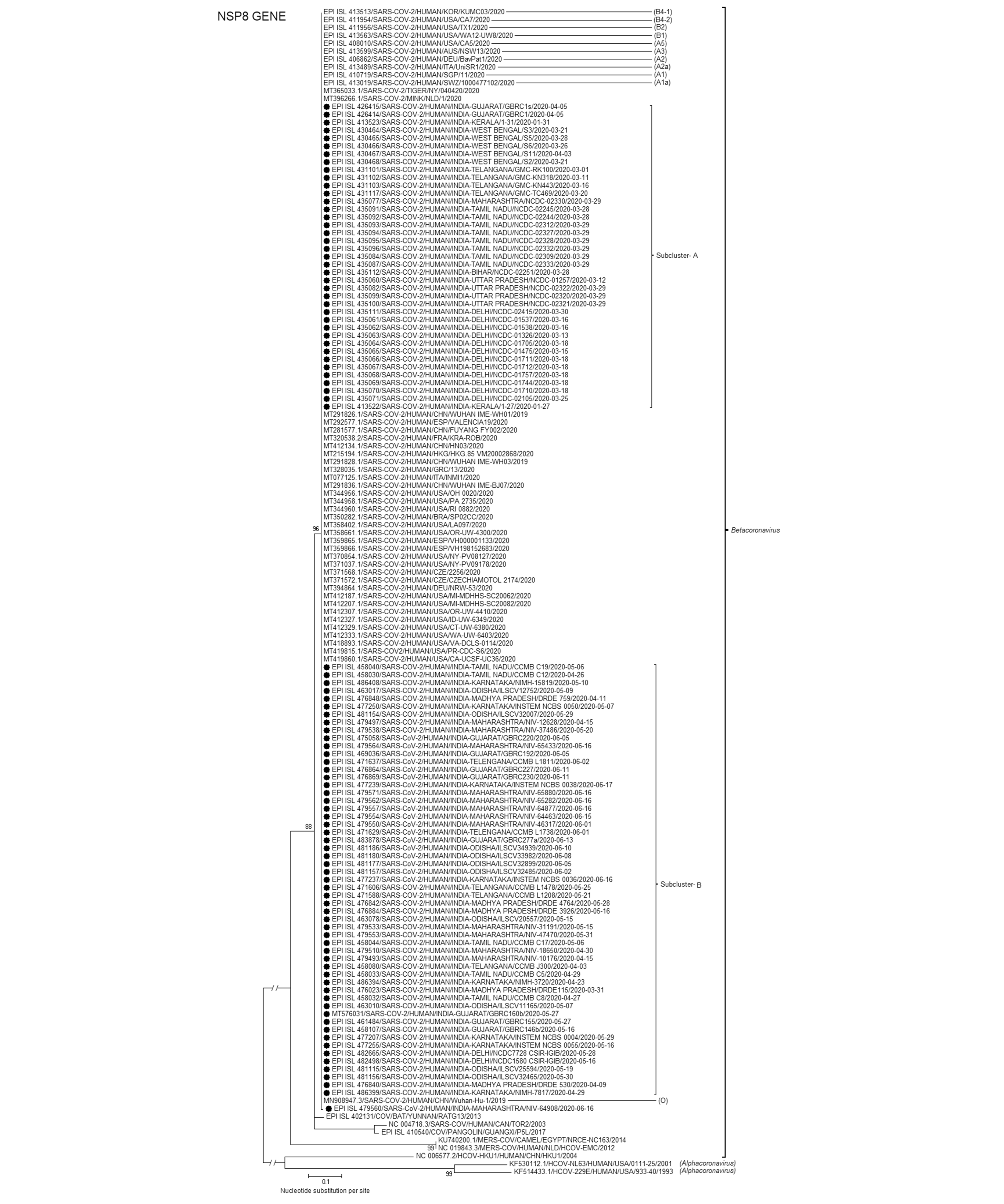

Supplement: Multimedia Appendix 9 [file bioinform_v1i1e20735_app9.png]

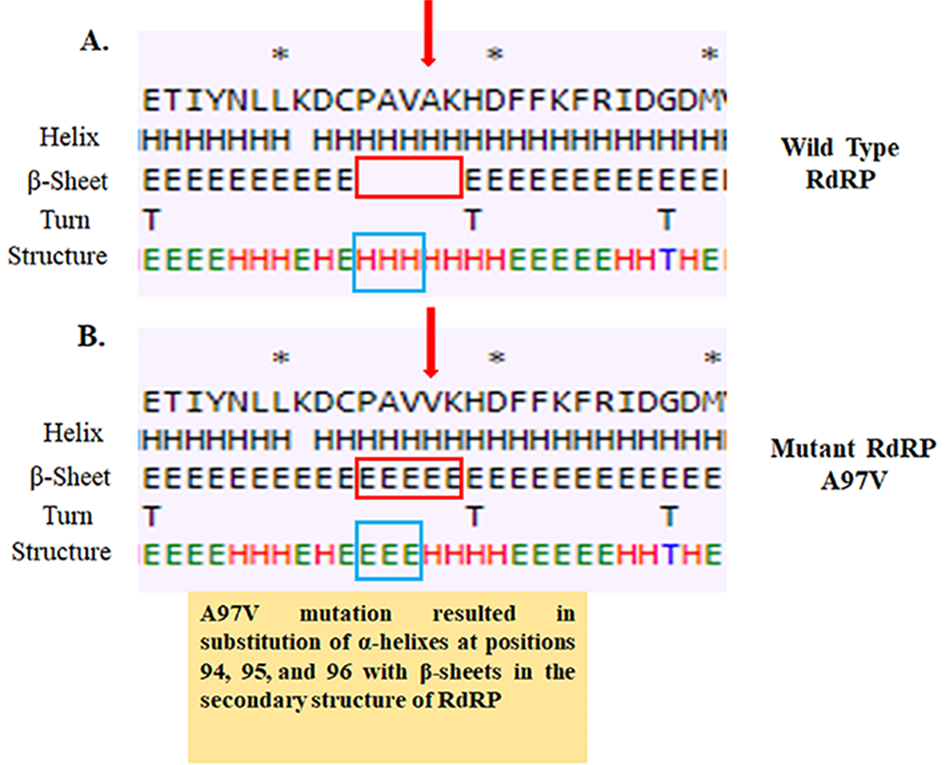

Supplement: Multimedia Appendix 10 [file bioinform_v1i1e20735_app10.png]
